# Impaired P2Y12 inhibition by clopidogrel in kidney transplant recipients: results from a cohort study

**DOI:** 10.1186/s12882-016-0270-2

**Published:** 2016-06-09

**Authors:** Clotilde Muller, Nathan Messas, Peggy Perrin, Jerome Olagne, Gabriela Gautier-Vargas, Noelle Cognard, Sophie Caillard, Bruno Moulin, Olivier Morel

**Affiliations:** Néphrologie-Transplantation, Nouvel Hôpital Civil, Centre Hospitalier Universitaire, Strasbourg, France; Pôle d’Activité Médico-Chirurgicale Cardiovasculaire, Hôpitaux Universitaires de Strasbourg, Nouvel Hôpital Civil, Université de Strasbourg, BP 426-67091 Strasbourg, France; Faculté de Médecine, Université de Strasbourg, Strasbourg, France; UMR 1109 Laboratoire d’Immunologie Rhumatologie, Université de Strasbourg, Strasbourg, France; UMR CNRS 7213 Laboratoire de Biophotonique et Pharmacologie, Faculté de Pharmacie, Université de Strasbourg, Illkirch, France

**Keywords:** Cardiovascular disease, Kidney transplantation, Platelet aggregation

## Abstract

**Background:**

Cardiovascular complications represent a major cause of morbidity and mortality for patients who received kidney transplantation (KT). However, the impact of KT and chronic immunosuppression on platelet response to clopidogrel in patients undergoing coronary or peripheral revascularization procedures remains unclear. This cohort study compares platelet responsiveness to clopidogrel as assessed byvasodilator-stimulated phosphoprotein (VASP) phosphorylation.

**Methods:**

The study population was divided between chronic kidney disease (CKD) patients who underwent KT (n = 36) and non-transplanted CKD patients (control group, n = 126). Patients were on maintenance antiplatelet therapy with clopidogrel 75 mg daily for at least 8 days. The mean platelet reactivity index (PRI) VASP values and the prevalence of high on-treatment platelet reactivity (HPR, defined as PRI VASP ≥61 %) were compared.

**Results:**

The mean PRI VASP value was significantly higher in the transplant group (60.1 ± 3 vs 51.2 ± 1.6 %; *p*=0.014). HPR was significantly more common in the transplant group on clopidogrel maintenance therapy (58 vs. 31 %; p = 0.011). KT was the only independent predictor of HPR (odds ratio: 2.6; 95 % confidence interval: 1.03–6.27, p = 0.03). The effect of treatment with calcineurin inhibitors on clopidogrel response could not be analyzed separately from the kidney transplant status.

**Conclusions:**

KT is associated with an increased prevalence of HPR. Our results suggest that plateletfunction tests may be clinically useful for the management of this specific population.

## Background

Chronic kidney disease (CKD) portends an increased risk of cardiovascular morbidity and mortality [1–7]. Moreover, a decreased glomerular filtration rate (GFR) has been associated with unfavourable clinical outcomes in patients undergoing percutaneous coronary intervention (PCI), possibly because of endothelial dysfunction and platelet activation [8–10]. Dual anti-platelet therapy with aspirin and P2Y12 receptor antagonists (including clopidogrel) is currently considered as a key strategy to reduce platelet reactivity and to prevent thrombotic complications following PCI [11, 12]. However, the clinical utility of clopidogrel may be hampered by the high inter-individual variability of inhibition of P2Y12-dependent platelet function. Accordingly, an elevated on-clopidogrel platelet reactivity (HPR) to adenosine diphosphate is a well-known risk factor for adverse events (especially stent thrombosis) following PCI [13–18]. In CKD patients, a significant and detrimental relationship between the severity of renal impairment and the extent of P2Y12 inhibition has been reported [19, 20]. Cardiovascular complications represent a major cause of morbidity and mortality for CKD patients undergoing kidney transplantation (KT) [21]. Notably, a previous investigation reported an association of clopidogrel use with a higher mortality risk, although caution should be exercised as it was an observational study with some data missing [22]. An increased platelet activation caused by calcineurin inhibitors has been advocated to explain the increased atherothrombotic risk observed in renal allograft recipients [23, 24]. However, the impact of KT and chronic immunosuppression on platelet response to clopidogrel in CKD patients undergoing coronary or peripheral revascularization procedures remains unclear. We therefore designed the current cohort study in order to compare platelet responsiveness to clopidogrel [as assessed by vasodilator-stimulated phosphoprotein (VASP) phosphorylation] in KT recipients and non-transplanted CKD patients.

## Methods

### Study population

Between November 2011 and January 2013, a total of 162 CKD patients undergoing coronary or peripheral stenting in our institution were retrospectively examined. Patients on maintenance antiplatelet therapy with clopidogrel 75 mg daily for at least 8 days were deemed eligible. Subjects with contraindications to antiplatelet therapy, platelet count <100,000/ml, or history of bleeding were excluded. Clinical interviews to assess adherence to antiplatelet therapy were systematically performed before inclusion. The study patients were divided into two groups, i.e., CKD patients who underwent KT (transplant group, *n* = 36) and non-transplanted CKD patients (control group, *n* = 126). Patients included in the transplant group were transplanted from March 1991 to December 2012. The reasons for transplantation were as follows: glomerulonephritis (*n* = 14), diabetic nephropathy (*n* = 9), genetic disease (*n* = 5), unclassified (*n* = 3), anomalies of the kidney and urinary tract (*n* = 2), and chronic interstitial nephritis (*n* = 2), and nephroangiosclerosis (*n* = 1). Patients who underwent KT received immunosuppressive drugs; specifically, calcineurin inhibitors and mycophenolate mofetil were given to 89 and 86 % of the study patients, respectively. Transplanted patients were on clopidogrel for more than 8 days (they were all on chronic therapy, introduced before or after transplantation) as in the control group. There was no patient follow up.

### Assessment of renal function

Baseline serum creatinine levels were determined in all participants at inclusion using isotope dilution mass spectrometry (IDMS), concomitantly with the platelet function tests. All samples were collected in the morning. The estimated glomerular filtration rate (eGFR) was calculated using the Modification of Diet in Renal Disease (MDRD) formula, as follows: eGFR = 175 × (creatinine (μmol/L) × 0.0113)^-1.154^ × age^- 0.203^ (×0.74 for women). CKD was defined as an eGFR <60 mL/min/1.73 m^2^. In the transplant group, results were based on the post-transplant renal function.

### Blood sampling and platelet function tests

Whole blood samples were collected by venipuncture into Vacutainer tubes (BD Vacutainer, Becton Dickinson, Sparks, MD, USA) containing 0.129 M sodium citrate and immediately sent to the hemostasis laboratory (EFS-Alsace) for the assessment of VASP phosphorylation status. Platelet VASP phosphorylation was determined with a standardized flow cytometric assay (Biocytex Platelet VASP kit, Marseille, France) as previously described [25]. Levels of VASP phosphorylation were expressed as the platelet reactivity index (PRI) calculated from the median fluorescence intensity of the samples incubated with prostaglandin E1 alone or prostaglandin E1 and adenosine diphosphate according to the following formula: PRI = [(median fluorescence intensity prostaglandin E1 – median fluorescence intensity prostaglandin E1 + adenosine diphosphate)/median fluorescence intensity prostaglandin E1]/100. The main advantages of the VASP assay include its selectivity for the P2Y12 pathway, its insensitivity towards glycoprotein IIb/IIIa inhibitors, the stability of the results for >24 h after blood sampling, and the interpretability of single measurements. We have previously shown by receiver-operating characteristic (ROC) curve analysis that 61 % is the optimal cut-off value for PRI in the prediction of adverse cardiovascular events in patients undergoing PCI (including those with CKD) [26]. Consequently, patients with a PRI VASP ≥61 % were considered to have a high on-clopidogrel platelet reactivity to clopidogrel (i.e., low responders).

### Pharmacodynamic endpoints

The following three pharmacodynamic endpoints were considered for this study: 1) analysis of platelet response to clopidogrel through the comparison of mean PRI VASP values in KT recipients and non-transplanted CKD patients; 2) prevalence of HPR (using a cut-off value for PRI VASP ≥61 %) in KT recipients and non-transplanted CKD patients; and 3) identification of the main predictors of HPR in the study participants.

### Statistical analysis

Continuous variables are expressed as means ± standard deviations (SD) and compared with the Student’s *t*-test (Gaussian data) or the Mann–Whitney *U* test (skewed variables). Categorical variables are presented as counts and percentages and compared with *χ*^2^ test. One-way analysis of variance was used for intergroup comparisons and calculation of trend tests. Univariate and multivariate logistic regression analyses were performed to identify the main predictors of HPR. Variables with a *p* value <0.20 on univariate analyses were entered in blocks into the multivariate model. Results are presented as odds ratios (ORs) with their 95 % confidence intervals (CIs). All computations were performed using the SPSS statistical software, version 17.0 (SPSS Inc., Chicago, IL, USA). Two-tailed *p* values <0.05 were considered statistically significant. When multiple comparison tests were performed, the statistical significance threshold was corrected using the Bonferroni’s method. Missing data were not replaced (deletion).

## Results

### Baseline characteristics of the study patients

A total of 162 CKD patients were included in the study. Thirty-six of them underwent KT (transplant group) and 126 were non-transplanted CKD patients who were not receiving immunosuppressive drugs (control group). All of the patients were being treated with clopidogrel 75 mg daily for at least 8 days. The baseline characteristics of the two study groups are summarized in Table 1. Patients in the transplant group were significantly younger (58.3 ± 1.6 vs. 72.6 ± 1.1 years, respectively; *p* < 0.001) and had a lower body mass index (BMI; 25.2 vs. 27.2 kg/m^2^, respectively; *p* = 0.05) than controls. The prevalence of diabetes was significantly higher in the control group than in the transplant group (58 vs. 39 %, respectively; *p* = 0.025). However, we did not find significant differences in eGFR between the two study groups (47.9 ± 3.7 mL/min/1.73 m^2^ in the transplant group vs. 39.5 ± 2.9 mL/min/1.73 m^2^ in the control group; *p* = 0.15). No difference was seen in platelet counts between groups. In the transplant group, 89 % of patients were being treated with calcineurin inhibitors, 86 % with mycophenolate mofetil, and 50 % with low-dose steroids.

**Table 1:** Baseline characteristics of the study patients

	Transplant group (*n* = 36)	Control group (*n* = 126)	*p*
Demographic data			
Age (years)	58.3 ± 1.6	72.6 ± 1.1	<0.001
Male sex, n (%)	26 (72 %)	79 (63 %)	0.33
Cardiovascular risk factors			
Diabetes mellitus, n (%)	14 (39 %)	73 (58 %)	0.025
Hypertension, n (%)	33 (92 %)	108 (86 %)	0.16
Current smoking, n (%)	14 (39 %)	60 (48 %)	0.12
Hypercholesterolemia, n (%)	30 (83 %)	104 (83 %)	0.15
Body mass index (kg/m^2^)	25.2 ± 0.8	27.2 ± 0.5	0.05
Biological data			
eGFR (MDRD formula) (mL/min/1.73 m^2^)	47.9 ± 3.7	39.5 ± 2.9	0.15
Anemia (Hb <12.5 g/dL), n (%)	17 (47 %)	68 (54 %)	0.47
CRP (mg/dL)	7.6 ± 1.5	25.4 ± 4.1	0.02

Data are expressed as means ± SD or counts (percentages), as appropriate. *Abbreviations*: *eGFR* estimated glomerular filtration rate, *MDRD* modification of diet in renal disease, *Hb* hemoglobin, *CRP* C-reactive protein

### Pharmacodynamic results: biological response to clopidogrel

**Fig. 1:**
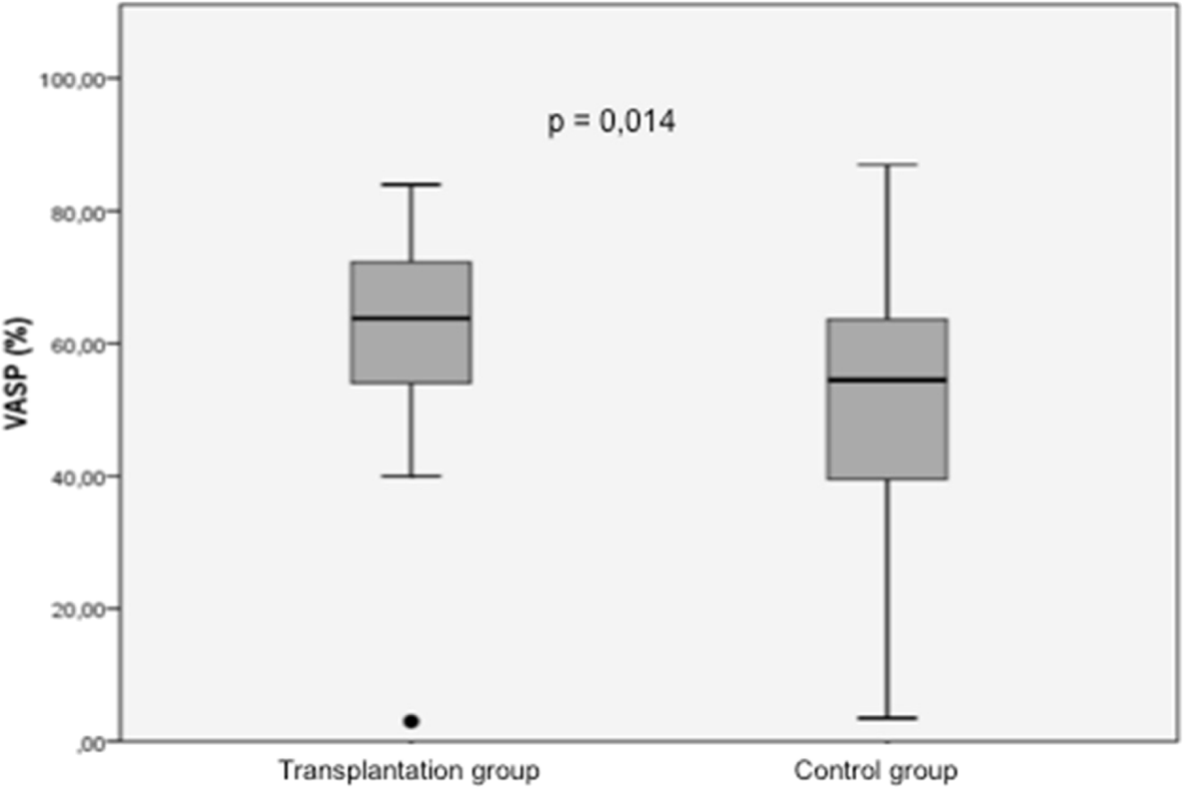
Mean PRI VASP values in the transplant group vs. in the control group. *PRI* platelet reactivity index, *VASP* vasodilatator-stimulated phosphoprotein

**Fig. 2:**
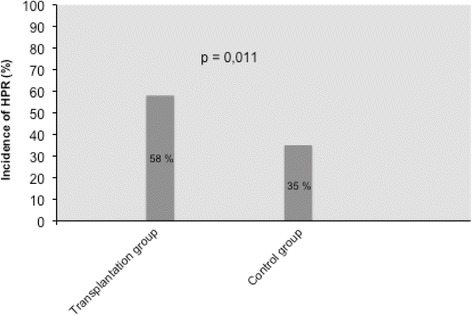
Prevalence of increased platelet reactivity (PRI VASP ≥61 %) in the transplant group vs. in the control group. *PRI* platelet reactivity index, *VASP* vasodilatator-stimulated phosphoprotein

The mean PRI VASP values in the two study groups are shown in Table 2 and Fig. 1. Compared with the control group, the mean PRI VASP value was significantly higher in the transplant group (60.1 ± 3 vs 51.2 ± 1.6 %, *p* = 0.014). The prevalence of HPR (PRI VASP ≥61 %) in the two study groups is reported in Fig. 2. HPR was significantly more common in the transplant group than in the control group on clopidogrel maintenance therapy (58 vs. 31 %, respectively; *p* = 0.011).

**Table 2:** Univariate logistic regression analysis for the prediction of high on-clopidogrel platelet reactivity (PRI VASP ≥61 %)

Variable	OR	CI (95 %)	*p*
Age (years)	0.98	0.96–1.00	0.15
Sex (male)	1.63	0.85–3.14	0.15
Diabetes mellitus	1.54	0.80–2.96	0.20
Body mass index (kg/m^2^)	1.08	1.01–1.15	0.02
Hypertension	1.94	0.72–5.26	0.19
Tobacco	1.18	0.63–2.22	0.61
Dyslipidemia	1.93	0.83–4.50	0.13
eGFR (mL/min/1.73 m^2^)	0.99	0.98–1.00	0.09
CRP (mg/dL)	0.99	0.97–1.00	0.09
Kidney transplantation	2.61	1.22–5.55	0.01
Calcineurin inhibitors use	3.15	1.41–7.02	0.005
Anti-metabolites use	1.94	0.87–4.31	0.11
Proton pump inhibitor use	1.15	0.58–2.29	0.69

*Abbreviations*: *PRI* platelet reactivity index, *VASP* vasodilator-stimulated phosphoprotein, *OR* odds ratio, *CI* confidence interval, *eGFR* estimated glomerular filtration rate, *CRP* C-reactive protein

### Predictors of high on-treatment platelet reactivity

Univariate logistic regression analysis demonstrated that advanced age, female sex, increased BMI, hypertension, dyslipidemia, high CRP values, low eGFR, and KT were significant predictors of HPR in patients on clopidogrel maintenance therapy (Table 2). After allowance for potential confounders, multivariate logistic regression analysis identified KT as independent risk factor for high on-treatment platelet reactivity (Table 3). Notably, KT was the only independent predictor of HPR (OR = 2.6; 95 % CI = 1.03–6.72; *p* = 0.04).

**Table 3:** Multivariate logistic regression analysis for the prediction of high on clopidogrel platelet reactivity (PRI VASP ≥61 %)

Variable	OR	CI (95 %)	*p*
Age (years)	0.99	0.96–1.02	0.48
Sex (male)	0.54	0.26–1.13	0.10
Body mass index (kg/m^2^)	1.71	0.74–3.97	0.21
Hypertension	1.68	0.57–4.98	0.35
Dyslipidemia	1.40	0.55–3.59	0.48
eGFR (mL/min/1.73 m^2^)	0.99	0.98–1.00	0.65
CRP (mg/dL)	0.99	0.98–1.00	0.24
Kidney transplantation	2.64	1.03–6.72	0.04

*Abbreviations PRI* platelet reactivity index, *VASP* vasodilator-stimulated phosphoprotein, *OR* odds ratio, *CI* confidence interval, *eGFR* estimated glomerular filtration rate, *CRP* C-reactive protein

## Discussion

This study highlighted three principal findings. First, we identified a high prevalence of low response to clopidogrel in CKD patients. Second, we observed an increased prevalence of high on-clopidogrel platelet reactivity in CKD patients who underwent KT. Finally, we identified KT as the only independent predictor of HPR in our study patients.

### Platelet response to clopidogrel in CKD patients: role of kidney transplantation

CKD patients have an increased risk of cardiovascular complications and represent a sizeable proportion of the population undergoing percutaneous coronary or peripheral revascularization procedures [1–7]. Several reports and registry studies focusing either on acute coronary syndromes or elective PCI have shown that CKD patients have higher rates of ischemic and post-procedural adverse events [27–29]. The mechanisms through which CKD may exert an unfavorable impact on clinical outcomes following coronary interventions are probably multifactorial (e.g., endothelial dysfunction, platelet activation, chronic inflammation, and presence of comorbidities). The exact role played by the impaired response to P2Y12 blockade in the pathogenesis of atherothrombotic complications among CKD patients remains unclear. Recent studies demonstrated that a decreased GFR is significantly associated with a poor response to thienopyridine therapy [26, 30]. We and others have also reported a significant interaction between CKD and high on-clopidogrel platelet reactivity (defined as a PRI VASP ≥61 %), with the latter parameter being a strong independent predictor of cardiac death [20, 31].

To the best of our knowledge, the magnitude of platelet inhibition by clopidogrel in CKD patients undergoing KT has not been previously investigated. The current study extends previous reports demonstrating a noxious interaction between KT and impaired platelet inhibition by clopidogrel. Herein, we have shown that a history of KT was an independent predictor factor of high platelet reactivity under clopidogrel treatment even after allowance for traditional risk factors for HPR (e.g., age, diabetes, or BMI). Notably, diabetes mellitus – a major factor for impaired platelet inhibition by clopidogrel – was significantly more common in non-transplanted CKD patients. Another important finding in the current study is the identification of an increased BMI as a risk factor for HPR. We and others have previously demonstrated the relevance of obesity as an independent predictor of HPR under clopidogrel treatment, regardless of the presence of diabetes [32, 33]. Because clopidogrel is metabolized in a lipophilic compound, we cannot exclude its diffusion within the adipose tissue, ultimately resulting in reduced circulating concentrations among overweight patients.

### Impact of immunosuppressive therapy on high on-treatment platelet reactivity in renal transplant recipients

Cardiovascular disorders continue to represent the leading cause of morbidity and mortality in CKD patients undergoing kidney transplantation [34, 35]. Observational registries have reported a 46-fold increase in cardiovascular mortality in this patient group compared with the general population [36, 37]. Besides conventional cardiovascular risk factors, chronic immunosuppression may play a significant role in increasing atherothrombotic risk both by promoting endothelial dysfunction and a chronic prothrombotic state. Accordingly, a recent study conducted on 62 KT patients suggested that calcineurin inhibitors may exert detrimental effects on both platelet and endothelial function. Specifically, chronic cyclosporine A and, in a lesser way tacrolimus administration may result in platelet activation and endothelial dysfunction, as there is an increase in parameters of platelet degranulation (CD62P), activation (PAC-1), platelet leukocyte aggregate formation (CD41 et CD11b) and inflammatory response (soluble CD40 ligand) [23, 24]. Similarly, treatment with mycophenolate mofetil has been shown to increase plasma concentrations of platelet activation markers (e.g., CD62) [38]. However, the exact pathophysiological mechanisms linking immunosuppression and platelet response to P2Y12 blockade remain unclear. The presence of interactions between immunosuppressive and antiplatelet medications cannot be excluded. Clopidogrel is a prodrug that requires hepatic conversion to exert its antiplatelet effects. Most of the absorbed clopidogrel (85 %) is hydrolyzed by an esterase to an inactive metabolite, whereas the remaining 15 % is rapidly metabolized by hepatic cytochrome P450 via a two-step process [39]. Because calcineurin inhibitors are similarly metabolized by cytochrome P450, it is possible that an increased hepatic metabolism may ultimately reduce the fraction of the active metabolite. Another possible explanation may involve transporter-based mechanisms (e.g., P-glycoprotein; P-gp). P-gp is expressed by enterocytes and mediates the ATP-dependent active transports of various drugs, including the intestinal efflux of clopidogrel. Notably, cyclosporine A may act as a potent inhibitor of P-gp [40]. We therefore speculate that the reduced response to clopidogrel observed in KT recipients may result from impaired drug absorption, altered metabolization, or both, ultimately resulting in lower levels of circulating active metabolites.

### Clinical perspectives

Because of sample size limitations, our study was not designed to assess the impact of HPR on hard clinical endpoints. We nonetheless believe that the high prevalence (up to 58 %) of impaired platelet inhibition to the 75 mg/day clopidogrel maintenance dose in KT recipients may be clinically relevant. Because HPR is common in renal transplant recipients, platelet function tests should be carefully considered for patients undergoing PCI, especially in presence of a positive history of atherothrombotic events despite dual antiplatelet therapy. Newer agents can be also used in patients who show significantly increased residual platelet aggregation following standard treatment. In this scenario, the potential advantages of the non-thienopyridine P2Y12 receptor antagonist ticagrelor (that does not require biotransformation) in KT recipients deserve further scrutiny, especially in presence of acute coronary syndromes. Notably, ticagrelor has been found to reduce mortality and the incidence of ischemic vascular events in a CKD population with an acceptable bleeding risk [41].

### Limitations

Some caveats of our study merit comment. Our observational registry has a single-center nature. Moreover, all laboratory results were from single-point measurements (a common limitation of studies assessing response to P2Y12 blockade). In the absence of analytical data, we cannot firmly establish the presence of reduced levels of clopidogrel active metabolite in CKD patients undergoing KT. The timing of VASP assessment was not uniform in the study patients. Moreover, we did not specifically compare the VASP assay with other platelet function tests (e.g., VerifyNow, platelet agreggometry). Concerning patients with high residual platelet reactivity, we did not specifically investigate the pharmacodynamic effects of higher clopidogrel dosages or novel P2Y12 inhibitors (e.g., prasugrel, ticagrelor). Because of sample size limitations, the current study was not adequately powered to draw firm conclusions on the potential impact of HPR on clinically hard endpoints. Because the great majority of KT patients were being treated with calcineurin inhibitors, we believe that the impact of therapy with calcineurin inhibitors on clopidogrel response could not be analyzed separately from the kidney transplant status.

## Conclusion

As a conclusion, KT is associated with an increased prevalence of high on-clopidogrel platelet reactivity. Our results suggest that platelet function tests may be clinically useful for the management of this specific population. However, the clinical impact and the precise mechanisms of this interaction remain to be established. Future studies should assess whether a tailored antiplatelet therapy based on PRI VASP monitoring may significantly improve clinical outcomes in CKD patients undergoing KT.

## Abbreviations

BMI, body mass index; CI, confidence interval; CKD, chronic kidney disease; GFR, glomerular filtration rate; HPR, high on-clopidogrel platelet reactivity; IDMS, isotope dilution mass spectrometry; KT, kidney transplantation; MDRD, Modification of diet in renal disease; OR, odds ratio; PCI, percutaneous coronary intervention; PRI, platelet reactivity index; SD, standard deviation; VASP, vasodilator-stimulated phosphoprotein
